# Supplementary figures and images for: The fast and the frugal: Divergent locomotory strategies drive limb lengthening in theropod dinosaurs
Source: PLoS One. 2020 May 13;15(5):e0223698. doi: 10.1371/journal.pone.0223698 (PMC7220109; doi:10.1371/journal.pone.0223698)

A

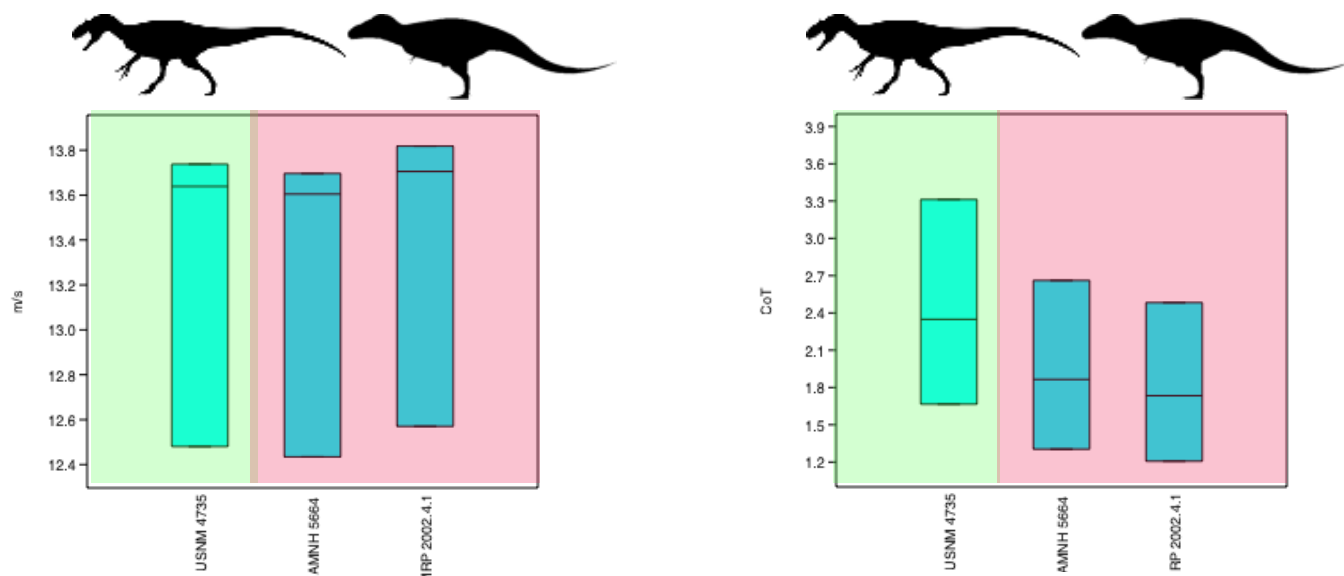

B

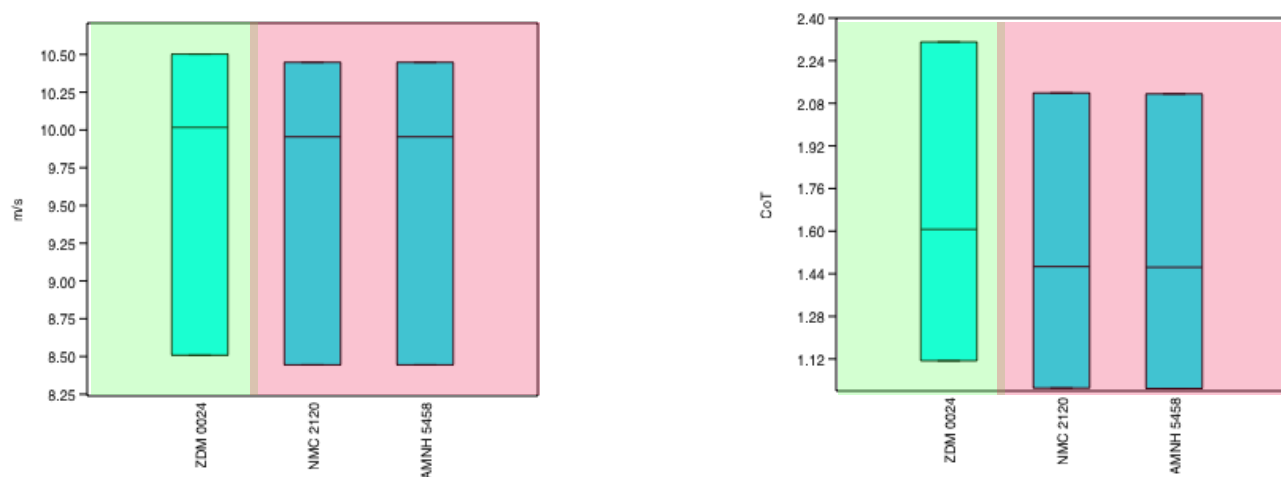

Supplement: S1 Fig — For all analyses green shaded box plots are basal theropods, red is tyrannosaurs. A) Smaller bodied specimens, mass range between 660-688kg, Ceratosaurs (USNM 4735) vs juvenile Tyrannosaurus rex (BMRP 2002.4.1) and Gorgosaurus (AMNH 5664). B) Midsized specimens, mass range between 2375-2430kg, Sinraptor (ZDM 0024) vs. Gorgosaurus (NMC 2120, AMNH 5458). For data see S6 Table. (PDF) [file pone.0223698.s007.pdf]

C

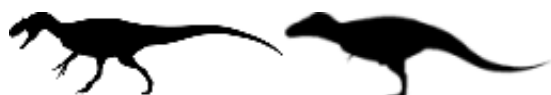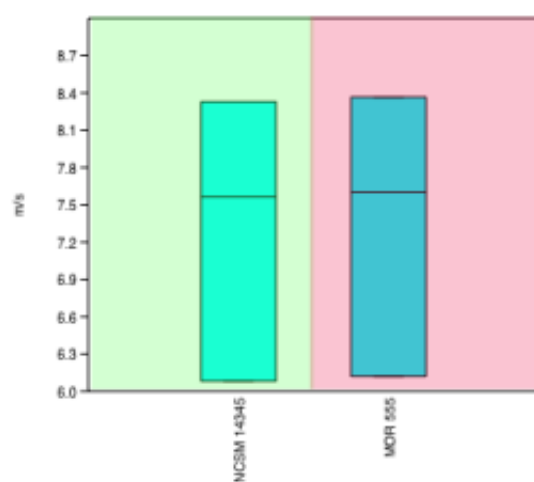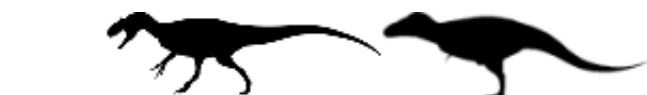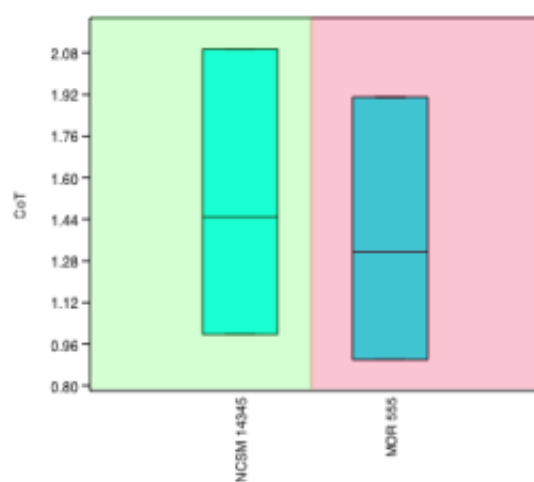

D

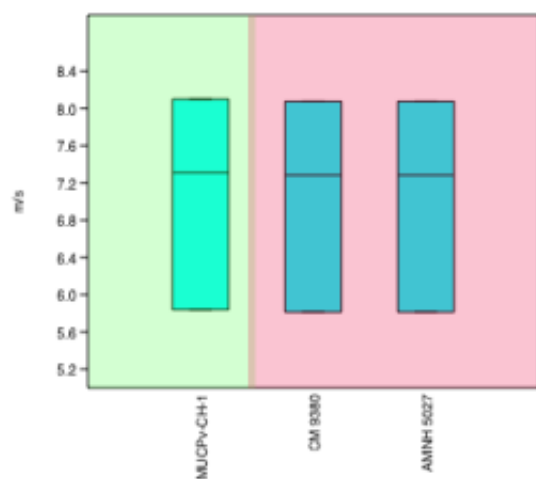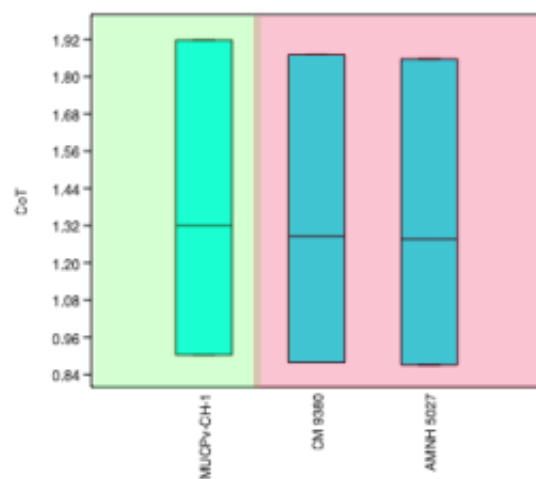

Supplement: S2 Fig — For all analyses green shaded box plots are basal theropods, red is tyrannosaurs. C) Large specimens, mass range between 6070-6170kg, Acrocanthosaurus (NCSM 14345) vs adult Tyrannosaurus rex (MOR 555) D) Largest specimens, mass range between 6900-7000kg, Giganotosaurus (MUCPv-CH-1) vs. adult Tyrannosaurus rex (CM 9380, AMNH 5027). For data see S6 Table. (PDF) [file pone.0223698.s008.pdf]
