# Supplementary material for: The fast and the frugal: Divergent locomotory strategies drive limb lengthening in theropod dinosaurs
Source: PLoS One. 2020 May 13;15(5):e0223698. doi: 10.1371/journal.pone.0223698 (PMC7220109; doi:10.1371/journal.pone.0223698)
Supplement: Attachment — Submitted filename: PONE reviewer response by Dececchi et al Dec 6th.docx [file pone.0223698.s010.docx]

**Response to reviewers' comments for manuscript PONE-D-19-26887:**

**“The fast and the frugal: Divergent locomotory strategies drive limb lengthening in theropod dinosaurs”**

Reviewer # 1 (Comments to the Author)

In the present study, the authors predict running speed and cost of transport in theropod dinosaurs. Unfortunately the study is not executed well. The manuscript is poorly constructed, and the narrative is extremely difficult to follow in many places. The grammar and legibility require considerable improvement, and the authors' non-scientific language in the conclusions is inappropriate. The justification for conducting the study is not entirely clear, and there does not seem to be any obvious hypotheses or research questions. The methodology requires the authors to bring in multiple numerical models from existing publications. However many of these predictive models are not explicitly laid out in the text, and the numerous assumptions associated with the models are not discussed. Holding posture as constant, for example, is a massive assumption given the considerable literature studying the postural shift occurring in the theropod lineage (Allen et al, 2013, Nature). Ultimately I was not sure what I was supposed to take away from the study. Is this a methodological study attempting to present a new metric for estimating running speed in fossils? Or rebut an existing one? Or is this a comparative functional morphology study looking at the evolution of running speed and cost of transport in the theropod lineage? If the purpose of the study was to rebut the Person and Currie 'distal hind limb index' by incorporating the parabolic model of Hirt et al, that would be a fine endeavour and would be achievable in 1500 words as a comment or reply to the original study. Unfortunately, in its present form, I do not believe this study is a meaningful contribution to the field of dinosaur palaeobiology.

We thank the reviewer for pointing this out. We have now corrected grammar and legibility throughout the manuscript. We have also modified the language and tone of the specific sections identified by the reviewer.

We respectfully disagree with the reviewer with regard to narrative. The various aspects of the multifaceted issue highlighted within the manuscript have been identified and addressed in sequence. The presentation sequence that we use, that is: 1. analyzing previous estimators of cursorial ability, 2. including the limiting factor of body size, and 3. evaluating a possible energetics explanation for the largest theropods as they exceed the size limitation of maximum running speed, is a necessary progression to show the audience why simple metrics alone cannot tell the entire ecological and paleobiological picture. This progression was specifically chosen because it highlights that central fact, that allometry and absolute mass have profound effects on paleoecology and energetics, and makes our arguments in the discussion and conclusions as accurate and clear as possible.

As part of our work, we included a section on examining how various simple metrics reflect these overarching behaviours (i.e., distal limb index of Person and Currie, 2016). This was not the central focus of our research. Nevertheless, our evaluation and rejection of the index proposed by Person and Currie (2016) as an accurate means to estimate highly cursorial and high speed runners amongst non-avian theropods is an important contribution to the overall literature, despite not being the major focus of this study. Its inclusion here is because it and other similar ratios are a central pillar of many studies, functional and phylogenetic , to infer behavioural repertoires . As such we felt if we did not include an analysis section on the reliability of this and similar metrics, which touch upon but is not central to the broader scope of work, our manuscript would be incomplete and less impactful.

With regard to justification and lack of a clearly stated hypothesis, while our justification is clearly stated in the opening lines of the abstract, and in the introduction in the original (lines 30-31 and 96-97 in the clean text) We have now added a hypothesis statement to the newest version of the manuscript to address the reviewer’s concern (lines 90 -93 in the clean text version).

Regarding the use of specific models in our study, we rely on modeling and mathematical estimations when performing any reconstruction. This is necessary in order to gain the prerequisite information to enter into our analysis. In this study, reliable, peer reviewed data sources were selected as a starting point and then modified as necessary to fit with the scope of our study. This strategy has been adopted in countless other peer-reviewed publications, including several by each of the main authors, where input data derived from other published sources is used. In these studies, even simple values such as linear measurements, which often differ slightly between left and right sides or between authors taking them, are constantly used as if they were a single fixed variable with no associated errors, and this is not commonly seen as a major flaw by the scientific community. All of these input equations and variables sourced have been appropriately referenced so that readers can refer to the procedures in the specific peer reviewed publications if required.

Regarding the reviewer’s objection to postural changes based on Allen et al. (2013), we argue that this study’s results do not invalidate or drastically impact our own for several reasons. Our study focuses specifically on running speed relationship with leg length and body mass, as well as how limb elongation alters the long term cost of transport in large theropods. Therefore, our results are not significantly affected by discussions of center of mass shifts within the Neoavian stem. At high speeds, such as what we are testing here since Froude numbers of 5 or greater are a fast run, large animals adopt a more upright stance, as seen in modern mammals at between 100-300kg (Bertram and Biewener, 1990; Biewener, 1990). Although smaller mammals remain proportionally more crouched, the challenge is to determine how significant this shift would be in the Theropods, especially as many small mammals often occupy niche spaces that include behaviours not suspected for non-avian taxa such as burrowing or climbing. We chose our crouching value, 0.8 of total limb length, and set it as a constant. This value was selected as it closely approximated that see in large (10+ kg) modern birds (Gatesy and Biewener 1991; Birn Jeffery et al. 2014; Bishop et al. 2018). While this value does vary somewhat in mid-stance allometrically in modern avians, but in taxa of around 1.0 kg and above this value, when you use just the three limb elements (Femur tibia and metatarsus) we used for non-avian theropods, is greater than 0.6-0.8 times hindlimb length (Gatsey and Biewener 1991; Bir-Jeffery et al. 2014; Bishop et al. 2018) in level running. Given that a) the vast majority of our taxa are above 1.0 kg (18/ 22 in the SVL dataset and 68/77 in the Leg only), and b) that there is a significant shift to a more crouched posture within Neornthines compared to the taxa investigated here (Allen et al. 2013), we defend this value as likely being within the range seen in non-avian theropods running at higher speeds. For our foraging cost calculations, this would be even less of an issue, as all individuals in our comparisons were estimated at minimally 660 kg, well within the range of upright stances using either a mammalian or avian model.

Beyond this, it is unlikely that the data from Allen et al. (2013) would significantly alter our analysis results as Allen et al. (2013) state that the origin of the “crouched’ posture occurs at Tetanurae which, in their phylogeny, is represented by *Dilophosaurus* and includes all taxa more crownward than the coleophysoids. In contrast, our analysis has only 5 (out of 93) members of the SVL dataset, and 5 (out of 77) in our ‘hindlimb only’ dataset that are more basal than this node. Thus, shifts in COM and posture occurring here should have little effect on the majority of our data. Furthermore, in the supporting information Figure 1 in Allen et al. (2013), they show very little dorsoventral shifting in COM observed between Theropoda and Ornithothoraces (nodes 5-14 in their phylogeny), and any significant alterations happens well beyond our taxonomic sampling. We feel that shifts in this axis, rather than cranial caudally, is much more important in determining hip height, especially during high speed running as examined here.

In addition, there are several other factors that make it unlikely that the COM shifts seen in Allen et al. (2013) are highly influential on the results presented here. First, we have some issues with the data Allen et al. (2013) used, including the fact that their cf. caenagnathus specimen (CM78001) listed by Allen et al. with a mass of between 9-16 kg, a pelvic limb of 0.539 m, and a femur length of 0.17m. This specimen is actually the type of *Anzu,* a fairly large oviraptorosaur. with a femur measuring 0.505 m, hindlimb length of around 1.2+ m, and a mass estimated at 200-300kg (Lamanna et al. 2014). Because of this discrepancy we suggest this data point is likely suspect, and as it comes directly before Eumaniraptora (and is the only species recorded for the node Maniraptora), that makes suggestions of any shifts that occur between Maniraptora and Eumaniraptora more uncertain. There are other issues, such as the mass estimate for *Archaeopteryx* being between 0.066-0.13kg while other volumetric methods have it well above 0.2 kg (Yalden 1983; Elzonwski 2002; Pontzer et al. 2009 amongst others), which effects the proposed shift amongst the basal most avians as well. This inaccuracy becomes important because, as stated in the supporting information for Allen et al. (2013), relative COM is related to body size. Proportional Caudofemoralis size also scales allometrically. This helps exaggerate any observed shift (especially if one removes the erroneously reconstructed cf. caenagnathus) from the basal maniraptoriform *Struthiomimus* between 394-741 kg to the eumaniraptorans *Velociraptor* at 9.9-18.3 kg. Of note, *Microraptor*, whose COM would be critical for understanding if this change is purely allometric or has some function significance amongst paravians, does not have a reconstructed caudofemoralis mass. Therefore, no discussion can be made on its behalf. One wonders whether any signals for a significantly different posture amongst derived maniraptorans compared to more basal tetanurans is mostly due to the size of the specimens selected. Allen et al. (2013) also does not include any complete small bodied more basal coelurosaurs such as *Compsognathus* or *Sinosauropteyrx,* thus any discussion of how stance amongst small bodied taxa differed , as well as any challenge to our use of metrics to reconstruct hip height and speed, is not possible.

Finally, as small bodied paravians possessed elongated leg feathers that would, if held at a lower angle such as a deep crouch, become damaged on the substrate during locomotion, this argues against them adopting the level of crouching seen in similar sized modern birds. We do have trackways from individuals likely well below 0.5kg in mass (Kim et al. 2018) without evidence of feather drags. While this does not prove that they must have had a more upright posture, it is suggestive that these taxa had proportionally higher stances and hip heights even in small bodied taxa. And even if we assume a crouched position during the run similar to modern birds of ~1 kg for our smaller bodied specimens, this would only reinforce our finding that hindlimb length is the limiting factor for maximum burst speed from them, as it would have further lowered these values below the level estimated using Hirt et al. (2017) mass based equation. This would only strengthen our argument for the selective value of increasing leg length amongst small (not large) theropods in order to become faster to avoid predators and secure prey.

Despite these arguments, we have included a new paragraph and analyses incorporated into Supporting Tables 3 and 4 that adjusts the posture of our specimens based on a regression from modern ground birds from the data of Gatesy and Biewener (1991). We would like to point out that this change offers only minor alteration to specimens below 40kg in body mass if we use their data. This includes the foot as part of the hindlimb (something we did not do for non-avian theropods), at which point the value shifts to 0.8 and thus we maintained that constant for all larger bodied specimens. More recent work by Bishop et al. (2018) using just the three leg elements we did to constitute “leg length” shows even less prevalent crouching levels in modern ground birds, with no specimen at or above 0.6kg having hip levels less than 80% of leg length. While there is an allometric effect it would suggest that, if anything, we are over estimating the level of crouch in larger taxa, something that would again only hasten the intersection point where body size precludes longer limbs having a positive effect on speed. That said, we did perform an adjustment in addition to our original data to incorporate crouching and this adjustment, while it does lower the absolute maximum speed attained, does little to the relative differences between individual specimens or clades discussed. Nor does it alter the overarching finding of a shift from a drive to maximize running potential in small to medium sized theropods, while reducing transportation costs dominate at larger size classes.

In a more general response to this reviewer, we believe that a paper combining the testing of existing estimators for cursoriality with evaluation of the major selective pressures driving increased leg length amongst different theropods clades, and evaluating the energetics of foraging amongst large bodied theropods, does make a substantial contribution to the paleobiological literature. The fact that we decided to combine several different, but related, analyses and datasets in a single manuscript is to us not a drawback, but a strength. We would therefore like to thank the reviewer for their comments, and would be keen to work with them on specific issues and sections that they deem as requiring more work. However, with respect, we do not see the foundation of the reviewer’s argument for rejection as being solid or providing any useful path forward in addressing this problem.

Reviewer #2: This paper examiness limb proportions and relates them to estimates of speed and cost of transport / foraging costs in non-avian theropod dinosaurs. They argue that limb proportions are not generally reliable for predicting speed (in large part because the equations do not take into account the effect of scaling at large body sizes), and that the long legs of large-bodied tyrannosaurids lowered the energetic cost of foraging compared to other large-bodied theropods.

I agree with the overall conclusions of the article. Limb proportions cannot be sufficient for speed estimates given the restrictions faced by large taxa. I'm not sure that many paleontologists disagree on this point these days, and I'm not sure that other researchers use 'cursoriality' to only mean top speed. It makes sense that proportionately long legs would make for better cost of transport, particularly given that, as far as I can tell, the equation being used is derived form Pontzer 2007 that only uses hip height.

I think the main weakness of the paper is that several of the fundamental equations are based on samples across animals that find overall trends based on log-transformed data. I don't disagree with these papers' conclusions- there is a relationship between size and absolute speed, and metabolic cost and hip height. However, using equations based on these big differences to examine fine-grained within clade differences is less reliable than more specific models. For instance, the Y scale in figure 3 shows that estimates from the mass limitation paper can be up to about double those based on Froude numbers. This combined with other factors, such as log-transformed data tending to produce visually tight clusters of disparate data at large values, and the lack of confidence interval/errors for some of these estimates, can produce precise-looking estimates and trends where it is unclear how confident we should be in these estimates. There may not be an applicable fix currently for this issue, but I do think that it might be worth reminding readers that even though regressions produce point estimates, that there is some confidence interval around that estimate that might blur otherwise clear-looking patterns.

I think that any paper that deals with cursoriality or speed and discusses theropods should include at least a brief discussion concerning gait and grounded running. Unfortunately, there is confusion in the literature about what "running" means even in papers dealing specifically with top speeds, and so it's best not to contribute to this confusion. Any equations derived from mammal data concerning speeds should also be defended given theropods ability to achieve running dynamics while maintaining dual support.

More detailed comments follow:

Line numbers should always be included in manuscripts.

We thank the reviewer for point this out, and have now added line numbers to the revised version.

The abstract seems a bit strong in regards to the impact of mass. Is body size really often overlooked when discussing speed in theropods? A few holdouts occasionally opine about Tyrannosaurus galloping but the serious literature hasn't bother with those sorts of ideas for a long while it seems to me. Also is cursoriality the equivalent of speed? It's not clear to me that Persons and Currie mean speed.

Re-reading Persons and Currie’s text, it is clear that they mean to evaluate running ability, and repeatedly reference maximum running speed, while noting that they really can’t test that using their proposed metrics. In addition, having listened to several talks by other members of this research group which all have used CLP score as a proxy for running speed in their taxon of interest. As for Tyrannosaurus top speed, even many serious works have postulated speed as being a major factor in their extended distal limb segments, even if they don’t think they ran at a gallop. Our finding that speed is highly limited by mass, not just in the largest of the large specimens but likely in all specimens over 1000kg, has a broader impact on our reconstruction hunting behaviour and paleoecology for theropods large, medium and small.

The variables in the supplemental tables should be described and explained somewhere.

We agree with the reviewer. These have now been listed in the description of Figure 3 as well as in the supporting tables, where they appear.

Throughout the manuscript interlimb is used when I think intralimb is meant. Given that the paper partially seems to be intended as a response to Persons and Currie, it is disheartening that Persons name is consistently misspelled as Parson throughout the text.

We thank the reviewer for pointing this out. We have now corrected this throughout the manuscript.

Figure 2- B is almost entirely redundant with A. Replot as one panel with Halszkaraptor receiving some unique symbol so that it can be picked out.

We agree with this comment, and have made the suggested modification.

"sharp demarcation" p15. The point estimates seems relatively distinct but there's no error on them. How much messier does it look when different estimates for each taxon are plotted? The next sentence reveals that estimates for one taxon can vary almost 3m/s.

With apologies for the confusion, we would like to clarify that this is the estimated speed across various speed estimator, and not the error. For example the comparison of *Changyuraptor* compared to *Sinosauropteyrx*, two species similar in SVL (2% difference) using any individual metric, we find that the former has a 131% higher estimated top speed than the later as show in Supporting Table 2. Thus, while the top speed estimate between metrics for any individual taxon may vary, and these are the different numbers the reviewer alluded to, the differences between individuals under the same estimator remain large. We have now added a sentence in the revised manuscript to clarify this point.

Table 3: B is confusing, needs to be explained or laid out better.

Is Figure 5 referenced in the text anywhere?

We thank the reviewer for pointing out this oversight, we have now added the figure reference in the revised manuscript.

I have made a few more notes in the attached pdf and have highlighted some obvious grammar errors.

We have now addressed these in the revised version where they occur.
