# Supplementary material for: The fast and the frugal: Divergent locomotory strategies drive limb lengthening in theropod dinosaurs
Source: PLoS One. 2020 May 13;15(5):e0223698. doi: 10.1371/journal.pone.0223698 (PMC7220109; doi:10.1371/journal.pone.0223698)
Supplement: Attachment — Submitted filename: Response to reviewers March 2020.docx [file pone.0223698.s011.docx]

Review Comments to the Author

Please use the space provided to explain your answers to the questions above. You may also include additional comments for the author, including concerns about dual publication, research ethics, or publication ethics. (Please upload your review as an attachment if it exceeds 20,000 characters)

Reviewer #1: Thank you to the authors for providing detailed and considered responses to our initial concerns. Through their inclusion of a new section on the potential confounding effects of body posture, I do believe the manuscript has been considerably improved. Likewise, the inclusion of a clear hypothesis has improved the framing of the study. Following up from Reviewer 2's previous suggestion, I do think two further additions are necessary in order for the research to be publishable:

1. Include confidence/prediction intervals at every step of the analysis. Although the authors are using volumetric mass estimates, many of those are published with an 'upper' and 'lower' bound solutions. Likewise, for any predictive model derived from empirical data (maximums speed equations of Alexandr or Ruiz and Torres, BMR estimates, CoT estimates from Pontzer), the specific model used should be clearly stated in text (including coefficients, r2 value etc) and 95% prediction intervals calculated. These can be easily calculated when the authors of the model have included their raw data (as is the case with Pontzer 2007, Grady et al etc.). Preferably, the authors should include this uncertainty as they progress through their analysis. This can be achieved through defining the 95% prediction interval and then running a random number generator within those bounds, say 1000 times. Progress those values on and repeat at the next stage of analysis. Ultimately, each species should then be represented by a distribution of values for predicted speed, CoT etc. This is a powerful approach, as it would then allow the authors to statistically test whether one species is different from another.

We agree with this suggestion, and in trying to accommodate it we have provided confidence intervals based on running speed estimates derived from Hirt et al. (2017) (based on 1.96x the standard error values provided in Supplemental Table 4) as well as cost of transport based on Pontzer 2007 which provides the equation used in Pontzer et al. 2009 (which does not provide data to determine error bars). It should be noted that neither Alexander 1976 nor Ruiz 2018 (this was an error on our part, for while Ruiz and Torices 2013 does talk about running and does correct the error in Alexander 1976 Ruiz 2018 directly address the application of this error to a theropod trackway and thus is more applicable for use on line 143. This has been corrected in the text) include confidence intervals nor standard error values. Furthermore papers that use these speed estimates such as Thulborn 1982, Mazetta and Blanco 2001, McCrea et al. 2014, Smith et al. 2016 (amongst others) calculate them without confidence intervals. Thus we estimated speeds from those equations based both on the data presented and in the way that is acceptable and commonly used in the field.

As for calculations from Froude numbers we do not include confidence intervals on those as the Froude number is a dimensionless parameter not a derived coefficient from a regression analysis and thus does not permit upper and lower bound estimates. For mass values, amongst the studies using volumetric reconstruction to estimate dinosaur size used in our analysis only those from Bates et al. (2009) include upper and lower bound estimates, the rest all include only a single value for calculated mass. Given that this represents only 2 data points in our analysis we do not believe that including them provides much added clarity or changes our results in any meaningful way. We have modified Figure 5 (include here as well) to document upper and lower bounds for the Hirt et al. 2017 speed values. We also calculated upper and lower bounds for CoT for the taxa included in Table 1and included these values as Sup. Table 6.

Besides the inability to gain the data to perform the analysis this reviewer request, another challenge is that we do not believe that their suggested approach is a) informative and b) impactful on our results. While it may appear to enhance the reliability of the data (though as we pointed out it is not possible to do given the sources that we used) its true value is limited. Biologically speaking those “randomly” selected points are not actually equal probabilistically. For example, without any prior knowledge of suspected phylogenetic signal in differences in metabolic costs (which for the tyrannosaurs and the more basal theropods used in this part of the analysis we have no evidence nor suggestion that there would be any) assuming that it is equally likely that one clade occupies the upper bounds while the other the lower is not justified. In order to show that our data suggests that there are genuinely marked CoT differences, but not speed differences we have included comparisons of the Upper and lower bounds estimates for similar sized taxa between these different groups included here and as Supporting Figures 1 and 2 in the manuscript. What is of note is that the for similar sized taxa the difference in speed values are minute, but CoT are much larger, often by close to an order of magnitude. For example the difference between top speed ( all data based on mass reconstructions from Snively et al. 2019, using the Hirt et al. 2017 speed limitation upper bounds) between Giganotosaurus (MUCPv-CH-1) and Tyrannosaur rex (either CM 9380 or AMNH 5027) is 0.02m/s or about 0.29% of top speed values (~8.9m/s) while the CoT differences are 2.4 and 3.1% higher for the basal theropods. We see similar values in smaller exemplars such as Sinraptor vs Gorgosaurs (both individuals at ~2400kg) where a speed difference of 0.05 m/s (0.5% of top speed) is met with a 8.3% difference in CoT. This pattern persists if one looks at the lower bounds or mean values across comparisons. We believe by including this data we can clearly show a distinct pattern of markedly higher savings in CoT compared to minor differences in top speed values across the comparisons. Given that CoT is also a cumulative value which effects the energy budget every step that animal takes during its lifetime, this is likely a much more impactful value for selection.

2. Following on from this, I would like to see the authors run their analysis on a few modern taxa to check the protocol produces sensible values. There are several volumetric models of extant animals available in the literature. Falkingham 2011 has a photogrammetry model of an elephant in the supplementary data, for example. I think the authors could have far more confidence in their results if they could demonstrate that they are capable of producing ballpark reasonable values for modern taxa for which we have the in vivo data.

We are slightly puzzled by this request as both the volumetric estimates we present as well as all speed and CoT equations used were derived from studies and protocols tested first on extant animals. Thus the reason we can have some confidence in these protocols is because they have been derived from living creatures. What we can say is that perhaps that our Froude estimated speed potential for the largest taxa (mass >6000kg) may be too high as the fastest a living elephant has reliably been documented to run is Fr=3.4 (Hutchinson et al. 2003). This may be a moot point as using Hirt et al.’s upper bounds speed values we find that these correspond to Froude values at around 2.5 for these extremely large theropods. This is similar too (but higher than) values seen in modern African elephants on level tracks (Hutchinson et al. 2003), conditions that are unlikely to occur in the environments these animals existed in. Thus again we are confident that these values to likely represent upper estimates of speed for these creatures.

Reviewer #2: Considering the revision and the authors' response, the authors do not seem to have addressed my largest two concerns.

The first is about the nature of the values calculated from the regressions. Consider, for example, the data from Table 2, where the authors argue for a clear difference between large bodied tyrannosauroids and other similarly sized theropods. Sinraptor and Giganotosaurus appear close to Tyrannosaur values, while Tarbosaurus is close to the Tyrannosaur values. Yes, the point estimates produce a pattern where Tyrannosaurs are generally below similarly-sized theropods but without an idea of the variance/error involved in estimation, it is unclear how strongly to interpret these results. I continue to believe that presenting point estimates for fossil taxa without any discussion or attempt to quantify variance, error, or confidence about these estimates is not nearly as helpful as if these were at included or at least considered. For instance, many of the large theropods exceed the largest taxon sampled in Pontzer's data (Obviously, since elephant is the largest available taxon with measured cost of transport data). Extrapolating beyond the sample set used to generate the regression is an issue that should be discussed in the paper. I think at least some discussion of these sorts of issues, even if the authors do not quantify them, is appropriate.

We addressed this issue above with the additions of new supporting tables and figures documenting more thoroughly the tyrannosaur values difference from other theropods of similar size. While we could include a small note about how the largest of our specimens extend beyond the modern taxa used to derived CoT equations unless there is reason to believe that one clade had a fundamentally different relationship between CoT and size than other how this would change our values. Even if our estimates for the largest theropods are off by 20% or more they should be off for all specimens by similar amounts, therefore not biasing our results one way or another and giving us the same overall conclusion (long legs are energetic savers in larger taxa) even if the caloric values we estimated are inaccurate.

The second is that in a paper dealing with theropods and using the word "running" the authors should make explicit what they mean by the term. This is not a kinematics or energetics paper, but it is very easy for other researchers and the public to misinterpret any results that deal with running speed without things being made clear.

The authors are confused as to why this is an issue for the reviewer, and what implications it has for this publications. Nevertheless we have included the following section in the text to make it clear that we are using “running ability” to mean more than overcoming the walk to run transition barrier.

“In extant vertebrates the walk to run transitions occurs at a Froude number > 1 [23], and a similar value is expected to hold for non-avian theropods with even the largest being suspected to achieve this feat [10, 11]. Following these parameters we define running ability here as the ability to achieve speeds corresponding to Froude numbers significantly higher than 1, as opposed to running capacity which is the ability to generate values of 1. We hypothesize that allometry will have significant consequences for running ability and that the selective weighting of top speed versus reducing energetic expenditures will vary across Theropoda concordant with changes in body size. We also hypothesize that amongst the largest theropods, > 1000kg, running ability, as assessed by top speed potential, will not be a significant factor in the influencing the relative level of elongation of the distal hindlimb, including in tyrannosaurs. “
